# Microstrip Antenna Design and Its Application in Moisture Detection

**DOI:** 10.3390/s26134291

**Published:** 2026-07-06

**Authors:** Shiqin Wang, Haotian Shi, Huichuan Lin, Huanting Chen, Jun Zeng, Zhimin He, Yan Li

**Affiliations:** 1 College of Physics and Information Engineering, Minnan Normal University, Zhangzhou 363000, China; wangshiqin547@163.com (S.W.); sth2025@mnnu.edu.cn (H.S.); lhc1810@mnnu.edu.cn (H.L.); cht1521@mnnu.edu.cn (H.C.); zj1838@mnnu.edu.cn (J.Z.); hzm0191@mnnu.edu.cn (Z.H.); 2 Optoelectronic Materials & Device Application Industry Technological Development Base of Fujian Province, Zhangzhou 363000, China; 3 Key Laboratory of Light Field Manipulation and System Integration Applications in Fujian Province, Zhangzhou 363000, China

**Keywords:** moisture content detection, new-type microstrip patch antenna, partial ground technique, corner truncation technique, optimization of antenna dimensions

## Abstract

**Highlights:**

This paper investigates the application of bandwidth broadening techniques to overcome the inherent limitation that the narrowband characteristics of conventional microstrip patch antennas fail to meet the requirements of moisture measurement. A broadband antenna featuring a simple structural design is developed, and a corresponding moisture detection system is established to evaluate the stability of the proposed system.

**What are the main findings?**

- Partial ground and corner truncation are used to overcome the narrowband limitation of microstrip patch antennas, resulting in a simple-structured wideband antenna.
- A reflective-type moisture detection system is developed utilizing a phase comparator.

**What are the implications of the main finding?**

- This work offers a novel approach toward achieving miniaturized and low-cost moisture detection.

**Abstract:**

Moisture content is a critical parameter during processing and storage. Although conventional microwave-based moisture detection methods enable rapid and non-destructive measurement, they are often hindered by high cost and susceptibility to design limitations and environmental fluctuations. In this study, a partial ground plane technique combined with a beveled partial ground and corner truncation topology is employed to enhance antenna architecture. By accounting for the nonlinear relationship between antenna dimensions and operating bandwidth, the bandwidth is significantly broadened without increasing the antenna footprint. A novel microstrip antenna is developed using low-cost epoxy resin (FR4) as the substrate. To address the instability and narrow bandwidth often associated with microstrip antennas, an electromagnetic simulation model was constructed using HFSS. This model characterizes the relationship between dimensional parameters and bandwidth, facilitating antenna optimization. Simulation results demonstrate that the operating frequency remains stable at 915 MHz, while the operating bandwidth is expanded from 0.91–0.92 GHz to 0.6–1.1 GHz without increasing the antenna size, thereby satisfying the requirements for material moisture detection. The proposed antenna is integrated into the moisture detection circuit developed in this study, and its stability is evaluated under various environmental conditions. Results indicate that the novel microstrip antenna achieves high detection performance and is robust against environmental variations, while offering reduced fabrication costs. This study provides a new direction for the miniaturization and cost reduction of moisture detection systems.

## 1. Introduction

Moisture content is a critical parameter affecting the physicochemical stability of materials during storage and transportation. In practical production and logistics processes, excessive moisture predisposes materials to mold, spoilage, or performance degradation, thereby compromising product quality and safety. Therefore, rapid and accurate moisture determination is of paramount importance [1,2,3]. Commonly used moisture detection methods include oven drying, Karl Fischer titration, near-infrared spectroscopy, and electrical impedance spectroscopy [4,5,6,7]. Among these, detection based on microstrip antennas, which utilize electromagnetic wave resonance characteristics, offers advantages such as non-contact operation, real-time online monitoring, and non-destructive testing. Microstrip antennas feature a planar structure with the benefits of simplicity, compact size, and light weight [8,9,10]. They enable indirect moisture measurement by detecting phase variations between transmitted and received signals to infer the dielectric properties of the material under test, thereby facilitating the determination of moisture content. However, their inherently narrow bandwidth limits the effective capture of moisture-related signals. Furthermore, broadening the bandwidth by increasing antenna dimensions compromises the advantage of compactness, rendering them less suitable for field installation [11]. Therefore, to meet the installation requirements of portable or online monitoring devices, it is essential to design a microstrip antenna that maintains structural compactness while reliably detecting material moisture under various environmental conditions. To this end, researchers have made significant contributions. Hou et al. [12] developed a non-destructive wheat moisture detection system based on a metasurface planar-wave antenna; however, the metasurface lens antenna employed in this system is relatively bulky. Liu et al. designed a horn lens antenna that transmits linearly polarized plane waves [13], enabling the measurement of dielectric properties of wheat in the 5.5–6.5 GHz frequency band. Nevertheless, the horn lens antenna used in this system is bulky and costly to fabricate. Julrat et al. designed a pair of low-cost printed Yagi–Uda antennas for measuring the dielectric properties of in-shell peanuts at 9.6 GHz. In their study, a rotating cubic sample container was employed to measure dielectric properties in six orientations; subsequently, a calibration equation correlating these properties with moisture content was established to achieve non-contact measurement [14]. Lewis and Trabelsi proposed a peanut moisture detection system using a conventional horn lens antenna based on the microwave free-space method [15]. This system employs three density-independent methods to estimate peanut moisture content at 5.8 GHz, effectively mitigating the influence of density on measurement accuracy. However, the antenna used in this system requires direct contact with the peanuts, and the experimental design did not account for the potential effects of antenna errors or container errors on detection accuracy. Florian Stern proposed a Vivaldi antenna array for soil moisture measurement [16], which significantly improves antenna gain and exhibits strong directivity; nevertheless, its large size limits its applicability to moisture detection in open environments. The team led by Sakol Julrat developed an open-ended rectangular substrate-integrated waveguide sensor on an FR4 substrate [17]. This sensor features a simple structure and ease of fabrication, making it suitable for integration onto conveyor belts for moisture detection of dynamic materials. Although the aforementioned methods can effectively measure the moisture content of grains, several challenges remain, such as bulky system size, high fabrication costs, and susceptibility of detection accuracy to environmental factors. Therefore, the design of a rectangular microstrip antenna that achieves both structural compactness and wide bandwidth is of great significance.

Therefore, this paper comprehensively considers the relationship between structural dimension parameters and operating bandwidth to design a novel microstrip antenna. First, the antenna structure is optimized using partial ground plane technology. Then, based on corner-truncation technology, the dimensional parameters are optimized with the goal of maximizing the operating bandwidth, resulting in a novel microstrip antenna that achieves both a compact structure and wide bandwidth performance. Finally, the proposed moisture detection system is experimentally validated to demonstrate its reliability and stability in material moisture content detection.

## 2. Structural Design of Microstrip Antenna

### 2.1. Microstrip Patch Antenna

A microstrip antenna is formed by affixing a thin conductive patch onto a dielectric substrate that is backed by a conductive ground plane. It is fed by a feeding structure such as a microstrip line or a coaxial cable, which excites a radio-frequency electromagnetic field between the conductive patch and the ground plane. Radiation is then emitted through the gap between the edges of the patch and the ground plane [18].

**Figure 1:**
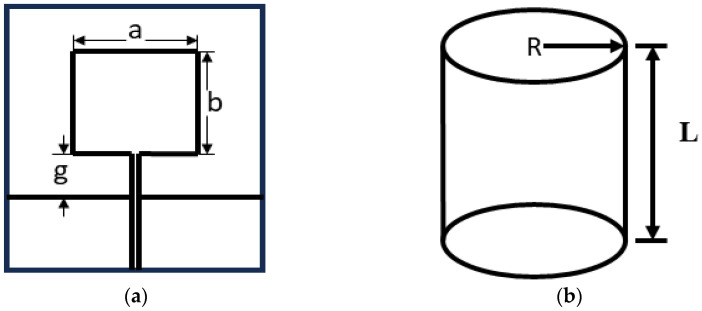
Microstrip structure. (**a**) Antenna prototype. (**b**) Antenna prototype and cylindrical structure.

A microstrip antenna adopts a multilayer structure comprising a radiating patch, a dielectric substrate, and a ground plane. To accommodate the surface of the material under test, epoxy resin (FR4) is selected as the dielectric substrate material in this study, with a relative permittivity of 4.4 and a thickness h of 0.5 mm. This dielectric substrate offers favorable electrical insulation, high breakdown field strength, and good ductility, thereby enhancing the flexibility and stability of the microstrip antenna in practical applications. Research indicates that at a frequency of 915 MHz, the dielectric loss factor (ε″) of the material exhibits a highly significant correlation with changes in moisture content. This strong correlation simplifies sensor calibration and improves measurement accuracy. For a rectangular microstrip antenna operating at a center frequency of f = 915 MHz [19], the planar monopole patch antenna can be approximated as a cylindrical structure of equivalent height L for estimation purposes. The geometry of the antenna patch is illustrated in Figure 1.

The resonant frequency of the antenna patch is directly determined by its geometric parameters. By cutting the cylindrical structure along its generatrix and flattening it, the resulting rectangle corresponds to the estimated rectangular radiating patch.

(1) 2πRL=ab

The input impedance of the monopole antenna is 36.5 + j21.25 Ω. The real part of the input impedance is slightly less than that of a quarter-wavelength monopole antenna, and the imaginary part exhibits inductive characteristics.

(2) l=0.24λF

where

(3) $$F=L/\left(L+R\right)$$

Thus, the following relationship can be obtained:

(4) λ=L+R0.24

The minimum operating frequency $f_{L}$ of the antenna is given by

(5) fL=Cλ=72/L+R

Considering the influence of the antenna gap g, the above formula is modified as follows:

(6) $$f_{L}=C/\lambda =72/\left(L+R+g\right)$$

Considering the influence of the substrate on the resonant frequency of the antenna, the above formula is modified to obtain

(7) fL=C/λ=72l+R+gεeff

where $\varepsilon_{eff}$ is the effective permittivity.

Assuming the relative permittivity of the substrate is $\varepsilon_{\tau }$, it can be roughly estimated as

(8) $$\varepsilon_{eff}=\frac{\varepsilon_{\tau }+1}{2}$$

Based on the above theoretical formulas for the patch antenna, the initial dimensions of the monopole patch antenna can be roughly estimated. In the subsequent design optimization process, further optimization can be performed using the electromagnetic simulation software ANSYS HFSS 19.1.0 (2018.1) based on these estimated initial dimensions to achieve the desired antenna performance.

### 2.2. Edge-Fed Microstrip Feeding

**Figure 2:**
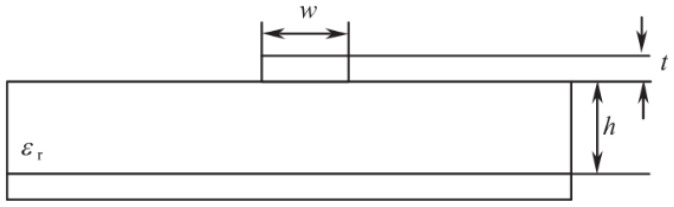
Microstrip structure.

Feeding is an indispensable part of antenna design. Microstrip line feeding is the most common feeding method, and the international standard characteristic impedance for transmission lines is 50 Ω. Given the width W1 and thickness t of the microstrip line, as well as the thickness h and relative permittivity $\varepsilon_{r}$ of the dielectric substrate, the structure of a microstrip feed line with a characteristic impedance of 50 Ω can be calculated using the relevant formula [20]. A schematic diagram of the microstrip line structure is shown in Figure 2.

The characteristic impedance of the microstrip line can be calculated using the following formula:

(9) z0=120π22πεr+1ln1+4hw′14+8εr11×4hw′+14+8εr1124hw′2+1+1εr2π2

where

(10) $$w^{\prime }=w+\Delta w^{\prime }$$

(11) Δw′=Δw1+1εr2

(12) Δwt=1πln4ⅇ/th2+1+πwt+1.12

By substituting the characteristic impedance of the microstrip line (50 Ω) and the antenna copper thickness (t = 0.016) into the above formula, the width of the microstrip feed line can be calculated, thereby achieving impedance matching.

## 3. Antenna Design

### 3.1. Design of the Microstrip Patch Antenna

**Figure 3:**
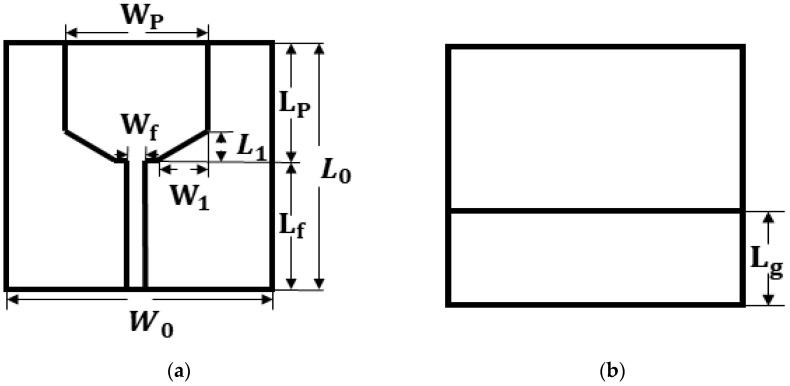
Schematic diagram of monopole patch antenna. (**a**) Antenna front view. (**b**) Antenna back view.

Conventional rectangular microstrip patch antennas primarily operate in the fundamental mode, such as TM_10_, and their impedance bandwidth is typically very narrow, usually in the range of 1% to 3%. This is because, near the resonant frequency, the antenna can be equivalently represented as a parallel RLC resonant circuit with a high Q-factor, resulting in an inherently narrow bandwidth. The operating frequency of such antennas cannot cover the bandwidth required for moisture measurement, necessitating parameter optimization of the patch antenna to broaden its relative bandwidth. This paper adopts the partial ground plane and corner truncation techniques, and simulation optimization is conducted using the electromagnetic simulation software HFSS. The rectangular microstrip patch antenna is truncated at the corners, and the dimensions of the truncated corners are optimized to achieve multi-frequency operation and bandwidth expansion. The constructed rectangular microstrip patch antenna is shown in Figure 3.

The parameters of the rectangular microstrip patch antenna are listed in Table 1. The antenna designed in this study utilizes an FR4 substrate with a relative permittivity $\varepsilon_{r}$ = 4.4 and a dielectric loss tangent tan ϭ = 0.030. The ground plane width is consistent with the substrate width, and the microstrip feed line has a characteristic impedance of 50 Ω.

### 3.2. Performance Parameters of the Antenna

**Figure 4:**
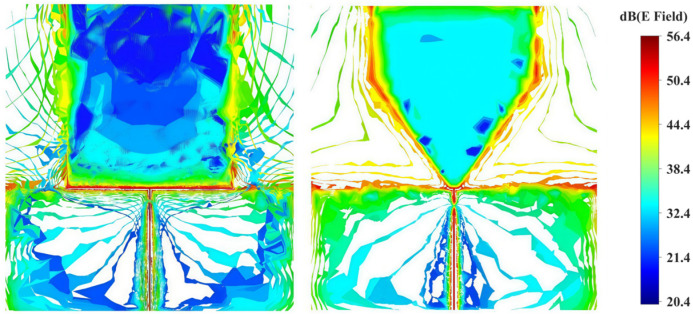
Schematic diagram of surface electric field distribution before and after corner truncation of the antenna.

The initial antenna was simulated using HFSS. Figure 4 shows the surface electric field distribution of the patch antenna before and after corner truncation. It can be observed that for the patch antenna, the surface current distribution flows along the edges of the antenna. In this study, the corner truncation method is employed to modify the structure of the antenna patch, thereby lengthening the path of the surface current along the patch edges. As a result, the effective length of the antenna is no longer constrained by its physical length and becomes greater than its physical length. The advantages of corner truncation are twofold. On one hand, it increases the effective length of the antenna, reducing the antenna size required for the corresponding frequency band. On the other hand, the specific truncated corner structure also influences the frequency band of the antenna by exciting higher-order modes, which, after integration, effectively broaden the operating bandwidth. At the equivalent circuit level, when a specific shape of corner truncation is fabricated on the antenna radiating patch, the surface current path is altered, causing it to flow tortuously around the truncated corner structure. This is equivalent to introducing a cascaded inductance into the antenna, which significantly affects the bandwidth and impedance matching of the antenna. In the subsequent sections, HFSS simulations are conducted to investigate the influence of the truncated corner dimensions on the S11 parameter of the antenna, aiming to determine the optimal antenna dimensions suitable for operation at 915 MHz.

**Figure 5:**
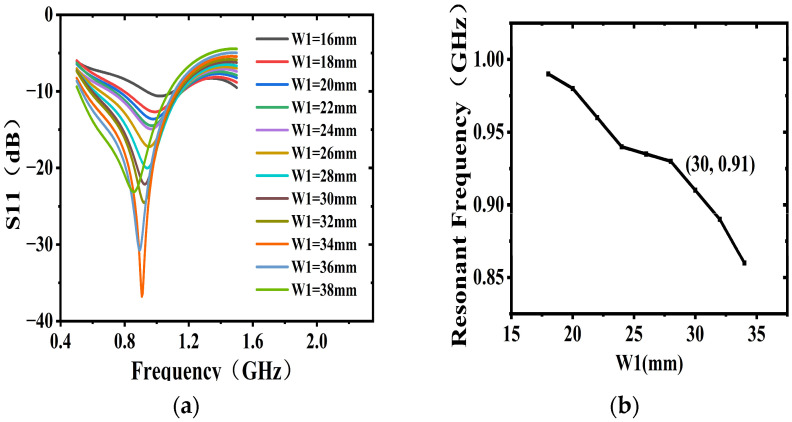
Schematic diagram of the influence of the corner truncation dimension W1 on the S11 parameter of the antenna. (**a**) Return loss plot. (**b**) Curve diagram of the influence of the corner truncation dimension W1 on the resonant frequency of the antenna.

Figure 5 illustrates the effect of the truncated corner dimension L1 on the S11 parameter of the antenna. It can be observed that as L1 increases, the notch depth of the S11 parameter becomes more pronounced, indicating improved impedance matching at the antenna’s resonant frequency, while the operating frequency shifts toward lower frequencies. When L1 is relatively small, a resonance is excited at a higher frequency due to the excitation of higher-order modes. Since the operating frequency of the moisture detection antenna in this study is required to be 915 MHz, it can be seen that when L1 = 48 mm, the resonant frequency is located at 915 MHz with S11 < −35 dB. The operational requirement for the S11 parameter is S11 < −10 dB. At the operating frequency, the impedance matching performance is satisfactory, and the antenna’s radiation capability meets the requirements.

**Figure 6:**
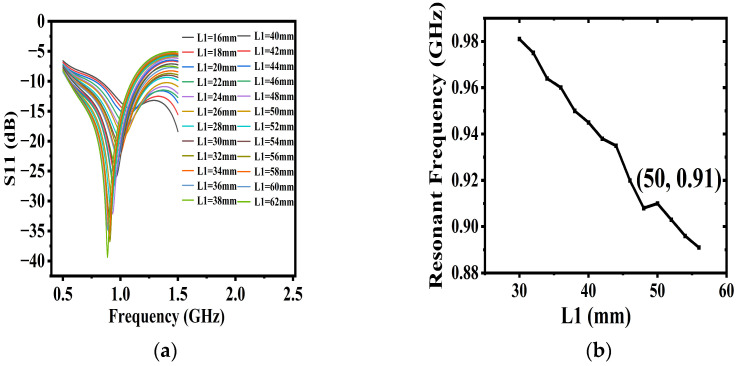
Schematic diagram of the influence of the corner truncation dimension L1 on the S11 parameter of the antenna. (**a**) Return loss plot. (**b**) Curve diagram of the influence of the corner truncation dimension L1 on the resonant frequency of the antenna.

Figure 6 presents the effect of the truncated corner dimension W1 on the S11 parameter of the antenna. It can be observed that, compared to W1, the dimension L1 has a relatively greater influence on the notch depth of S11. As W1 increases, the resonant frequency decreases, and the reflection coefficient exhibits noticeable fluctuations. Without affecting the resonant frequency, it can be seen that at the operating frequency of 915 MHz, when W1 = 34 mm, the reflection coefficient satisfies S11 < −35 dB. When W1 is relatively small, the low-frequency resonance gradually disappears because the antenna excites higher-order modes and resonates at these higher-order modes.

**Figure 7:**
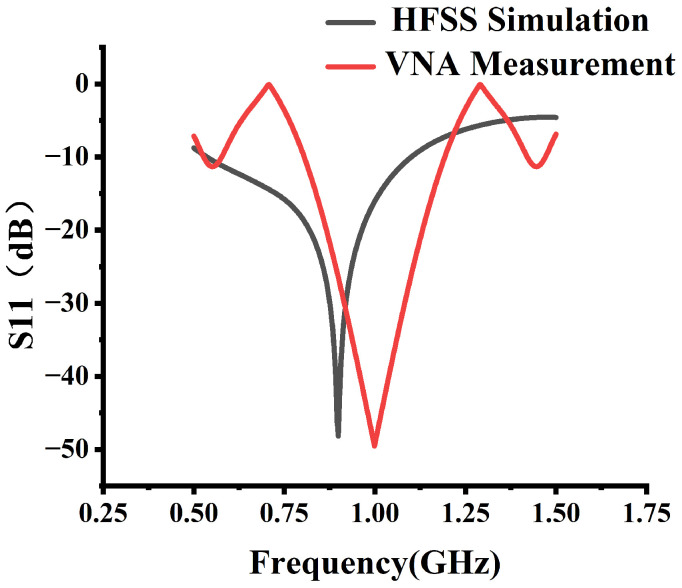
The return loss of the antenna.

The antenna with the aforementioned parameters and structure was simulated and analyzed using HFSS software, and the relationship between frequency and return loss is shown in Figure 7. It can be observed from the curve in Figure 7 that the antenna achieves S11 < −10 dB over the frequency range from 0.5 GHz to 1.1 GHz, indicating that it is a wideband antenna covering the operating frequency of 915 MHz. The absolute bandwidth reaches 0.6 GHz, and the relative bandwidth exceeds 65%. In contrast, conventional patch antennas typically achieve a relative bandwidth of only about 5%. Corner truncation is applied to the antenna in this work, significantly broadening its relative bandwidth. This is particularly important for low-frequency antennas such as those operating at 915 MHz, where the relative bandwidth is inherently narrow, making normal operation challenging. Through corner truncation and dimensional optimization, the antenna successfully fulfills the signal transmission and reception functions required for the moisture detection system.

To quantitatively evaluate the discrepancies between simulated and measured results, key parameters were extracted from the S11 curves shown in Figure 7. The simulated resonance frequency is 0.915 GHz, while the measured value shifts to approximately 1.00 GHz, representing an absolute shift of +0.085 GHz and a relative deviation of approximately 9.3% with respect to the center frequency. In terms of S11 magnitude, the simulated resonance depth reaches −35 dB, whereas the measured resonance depth is approximately −18.5 dB, yielding a difference of 16.5 dB. The simulated −10 dB impedance bandwidth is 0.60 GHz (covering 0.50–1.10 GHz), while the measured bandwidth is 0.50 GHz (covering 0.55–1.05 GHz), representing a narrowing of approximately 0.10 GHz. Nevertheless, the measured bandwidth still fully covers the operating frequency of 915 MHz, where the measured S11 is approximately −12 dB, satisfying the normal operation requirement for the antenna. In addition, ripples of approximately ±1.2 dB are observed around 0.5 GHz and 1.4 GHz in the measured curve, which are not present in the simulation.

These discrepancies can be attributed to the following quantitative factors. First, the simulation assumes a permittivity of 4.4 for the FR4 substrate, whereas the actual material exhibits a tolerance of approximately ±0.2; theoretically, a change of 0.1 in permittivity introduces approximately 1% frequency uncertainty. Second, the antenna was fabricated using a wet etching process with an etching accuracy of approximately ±0.05 mm, which contributes an estimated 0.5–1% frequency shift. These two factors together constitute the main causes of the observed frequency shift. In addition, variations in the FR4 loss tangent (assumed as 0.02 in the simulation) and substrate thickness tolerance (0.5 ± 0.05 mm) collectively contribute approximately 1–3 dB of S11 magnitude uncertainty. Furthermore, the soldering quality and parasitic effects of the coaxial-to-microstrip connector were not fully modeled in HFSS, directly contributing to the ripples of approximately ±1.2 dB. Finally, the finite ground plane and surrounding objects in the measurement environment may introduce coupling and radiation effects not present in the simulation. The accumulated effect of these error sources is estimated to yield a total frequency uncertainty of 2–3% and an S11 magnitude uncertainty of 2–4 dB, which is consistent with the observed simulation–measurement discrepancies.

Despite these discrepancies, the fabricated antenna operates properly at 915 MHz with a measured S11 of approximately −12 dB (below the −10 dB threshold) and a gain of approximately −2 dB, which provides sufficient received signal power for the AD8302 phase detector (sensitivity threshold: −60 dBm), confirming that the antenna can successfully fulfill the signal transmission and reception requirements for the moisture detection system.

**Figure 8:**
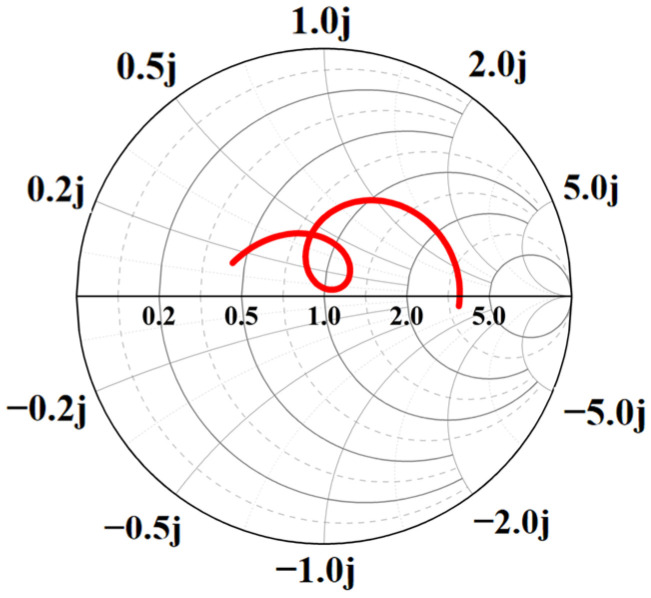
Smith chart of the antenna within the target frequency band.

Figure 8 presents the Smith chart trajectory of the designed antenna within the target frequency band. It can be observed that at the target resonant frequency of 915 MHz, the trajectory is located very close to the center of the Smith chart (the 50 Ω point). At this frequency, the input impedance of the antenna is approximately Zin ≈ 50 + j0Ω, achieving good conjugate matching with the source impedance. This observation is consistent with the deep notch in the S11 parameter at 915 MHz, as shown in Figure 7. The tight loop of the trajectory around the center point further indicates that the antenna exhibits a low voltage standing wave ratio (VSWR) and high radiation efficiency at this frequency.

**Figure 9:**
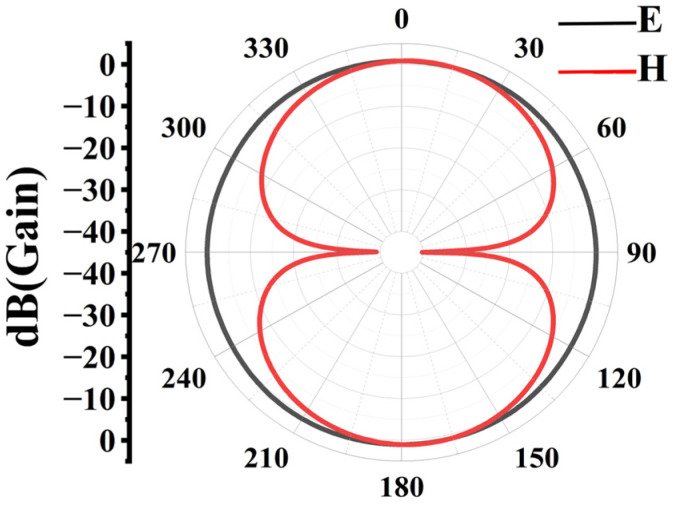
Directional gain diagram of the antenna.

**Figure 10:**
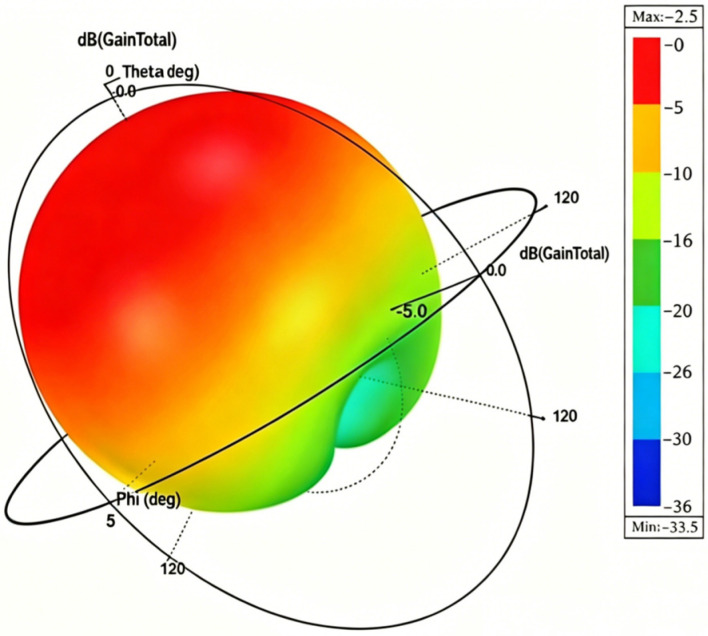
3D directional gain diagram of the antenna.

Figure 9 shows the E-plane and H-plane gain patterns of the antenna at 915 MHz, Figure 10 is the corresponding 3D gain plot. The H-plane pattern is figure-eight-shaped, while the E-plane pattern is nearly omnidirectional, indicating good radiation and gain performance with a maximum gain of −2.0 dB. In this humidity detection system, the distance between the test sample and the antenna is very short (<10 cm), and a reflective phase detection architecture is used. As specified in the AD8302 datasheet, even with −2 dB antenna gain, the received signal power is still far above the phase detector’s sensitivity threshold (typically −60 dBm). Hence, the −2 dB gain does not limit system detection. Moreover, in such near-field coupling sensing applications, excessively high gain may introduce environmental reflections that interfere with phase measurement.

It can be observed that the antenna design structure proposed in this paper not only effectively broadens the operating bandwidth but also does not significantly affect the gain characteristics. The corner truncation operation is simpler than conventional complex designs such as slotting or perforation, effectively reducing the fabrication difficulty and cost of the antenna.

### 3.3. Necessity of Broadband Antenna Design in Single-Frequency Detection Systems

In a permittivity sensing system based on phase comparison, the antenna typically operates at a single fixed frequency. In this work, an operating frequency of 915 MHz is adopted. Under such circumstances, the impedance bandwidth of the antenna is often considered sufficient as long as it covers this single frequency point, making a broadband design seemingly unnecessary. However, this section will demonstrate that in applications where the dielectric environment changes dynamically due to material loading, a broadband antenna becomes critical for maintaining the robustness of the system measurement, rather than being a redundant design.

The operating frequency of an antenna satisfies the following approximate relationship with the effective relative permittivity $\varepsilon_{r,e_{ff}}$ of its surrounding medium:

(13) $$f_{r}=\frac{1}{\sqrt{\varepsilon_{r,e_{ff}}}}$$

In moisture detection, when the moisture content of a material varies from 0% (εᵣ ≈ 3) to 20% (εᵣ ≈ 15), the effective permittivity around the antenna changes significantly. This variation causes a downward shift in the antenna’s resonant frequency. For a typical narrowband antenna (e.g., an antenna originally designed to operate at 0.91–0.92 GHz), its relative bandwidth is only 1.1%. When material loading induces a frequency shift exceeding 50 MHz, the input impedance of the antenna at 915 MHz becomes severely mismatched, and the radiation efficiency drops sharply, leading to a significant deterioration in phase detection accuracy. Therefore, the broadband design essentially provides a frequency “safety margin,” ensuring that the antenna maintains stable radiation performance and impedance matching at the fixed operating frequency despite variations in the dielectric environment.

**Figure 11:**
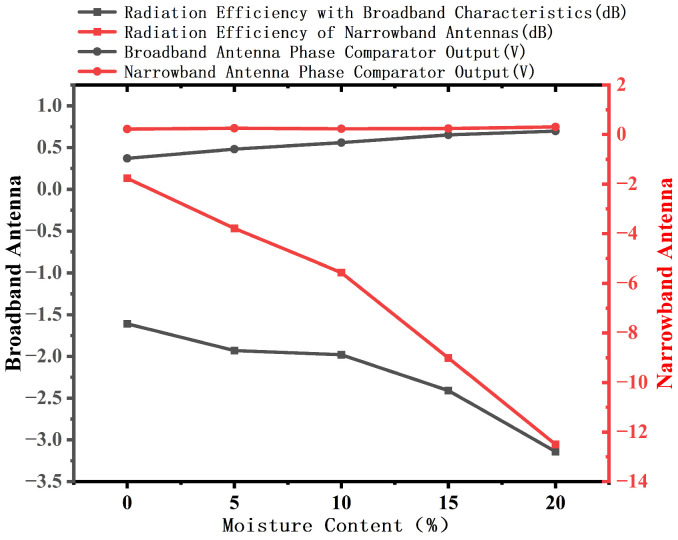
Schematic comparison of gain and output voltage between broadband and narrowband antennas for moisture measurement.

To validate the above mechanism, a comparative experiment was carried out. The proposed broadband antenna and a typical commercial narrowband antenna (operating at 0.91–0.92 GHz) were separately placed in rice samples with varying moisture contents (0%, 5%, 10%, 15%, and 20%). Following the experimental protocol described in Section 4, the radiation efficiency and the output voltage of the phase detection circuit were measured at a fixed operating frequency of 915 MHz. The experimental results are shown in Figure 11.

From the experimental results, it can be observed that within the material moisture range of 0% to 20%, the phase output voltage of the broadband antenna varies by 0.30 V (from 0.30 V to 0.60 V), whereas that of the narrowband antenna fluctuates only within a range of 0.2 V. The sensitivity of the former is significantly higher than that of the latter. The narrowband antenna is almost incapable of detecting changes in moisture content. The physical origin of this difference lies in the distinct impedance-phase response characteristics of the two types of antennas. For the narrowband antenna, the impedance varies sharply near the resonance frequency. Once detuned due to an increase in permittivity, the reflection coefficient phase enters a relatively flat region, rendering the phase output insensitive to material variations. In contrast, the broadband antenna exhibits a wide and flat impedance characteristic, ensuring that the fixed operating frequency of 915 MHz always falls within a region of steep phase slope. This effectively converts dielectric environmental changes into variations in the phase output voltage. Meanwhile, the broadband antenna maintains a stable radiation efficiency (from −1.0 dB to −3 dB) across the entire humidity range, while the radiation efficiency of the narrowband antenna deteriorates to −12.5 dB at 20% moisture content.

In summary, the core advantages of the broadband antenna in a single-frequency phase detection system are twofold: it offers higher phase sensitivity to dielectric environmental variations at a fixed frequency, and it possesses excellent detuning immunity. These characteristics make it a superior choice for moisture detection systems based on permittivity sensing principles.

## 4. Measurement and Testing of the Antenna in a Microwave Moisture Detection System

**Figure 12:**
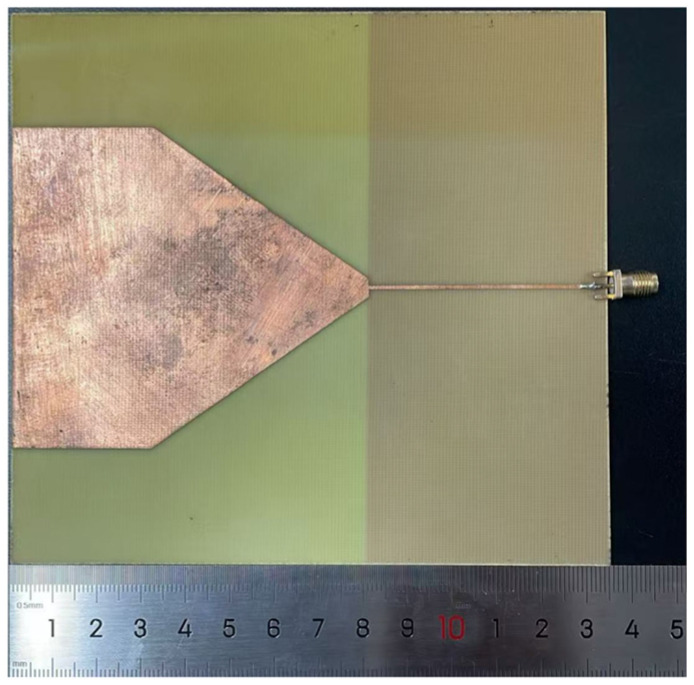
Physical diagram of the 915 MHz patch antenna.

Based on the dimensional parameters of the novel microstrip antenna, a prototype was fabricated using flexible FR4 as the dielectric substrate and copper as the conductive material, as shown in Figure 12. To evaluate the bandwidth performance of this novel flexible microstrip antenna and its capability for material moisture detection, moisture detection experiments were conducted under various environmental conditions.

### 4.1. Microwave Moisture Detection Test

**Figure 13:**
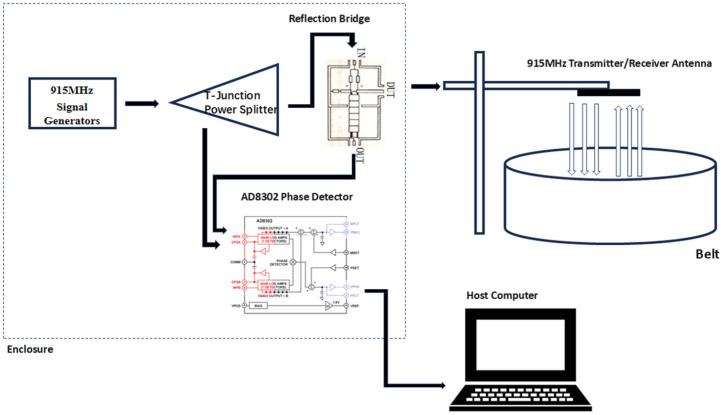
Hardware component diagram of the moisture analyzer system.

Studies on liquid characterization using microwave methods can be found in the literature [21,22,23]. However, the proposed solutions are not suitable for routine moisture detection, primarily due to the very small volume of material exposed to the electromagnetic field, as well as the large size, poor integrability, and high cost of the antennas and detection equipment. To evaluate the performance of the novel microstrip antenna in detecting actual moisture-related signals, a moisture detection experimental setup is constructed in this study to investigate the effect of materials with different moisture contents on the signal within the detection system. First, a radio-frequency signal source generates a 915 MHz sinusoidal wave signal, which is radiated into free space via the antenna. When the signal encounters the material under test, reflection occurs, and the reflected waveform is received by the same antenna. Since the dielectric constant of water is significantly higher than that of other materials, it affects the propagation velocity of electromagnetic waves, thereby altering the phase difference between the transmitted and received signals. By comparing the phase difference between the incident and reflected waves, the characteristics of the medium can be obtained [24], as shown in Figure 13.

The experimental setup mainly comprises a 915 MHz signal source, a power divider, a bridge, a phase comparator AD8302 was sourced from Analog Devices, Inc., Wilmington, MA, USA, the STM32 microcontroller was sourced from STMicroelectronics, Geneva, Switzerland. In the experiment, a signal generator is used to produce a single-frequency sinusoidal wave signal at 915 MHz. This signal is split into two in-phase signals by the power divider: one is directly fed into the AD8302 phase comparator as a reference signal, while the other is fed into the novel microstrip antenna via a T-junction bridge and radiated toward the material under test. The signal reflected from the material under test is received by the same antenna and subsequently input into the AD8302 as the measurement signal. The phase comparator outputs a corresponding voltage value based on the phase difference between the two signals [25]. This voltage is acquired by the STM32 microcontroller and displayed in real time on a capacitive touchscreen. The STM32 microcontroller acquires the AD8302 output voltage 50 times per second and outputs the average value as the final result. In the experiment, to evaluate the stability of the antenna, the effects of different environmental conditions on the material under test with the same moisture gradient are investigated, including variations in ambient temperature and the distance between the antenna and the material under test.

In the application of microwave-based moisture detection, the influence of ambient temperature T and the distance d between the antenna and the material under test on the phase difference Δϕ can generally be summarized by the following functional relationship:

(14) $$\Delta \phi =f\left(M,T,d\right)=\frac{4\pi d}{\lambda }\left(\sqrt{\varepsilon_{r}^{\prime }\left(M,T\right)}-1\right)+\phi \left(T\right)$$

where Δϕ is the phase difference measured by the phase comparator AD8302 (unit: rad); M is the moisture content of the material under test (%), which is the core variable to be measured; T is the ambient temperature (°C); d is the distance between the antenna and the material under test (m); λ is the wavelength in free space (m), determined by the signal frequency f = 915 MHz, i.e., λ = c/f, where c is the speed of light; $\sqrt{\varepsilon_{r}^{\prime }\left(M,T\right)}$ is the real part of the relative permittivity of the material under test at temperature T, which is a function of moisture content M and temperature T, reflecting the variation of the material’s dielectric properties with temperature and moisture; and ϕ(T) is the additional phase term introduced by the temperature drift of the system (including the antenna, the circuitry, and the AD8302 itself). Although the AD8302 integrates matched logarithmic detectors to suppress temperature drift, its residual temperature dependence still needs to be considered in practical applications [26].

To better evaluate the detection performance of the novel microstrip antenna, this study conducted moisture detection experiments under two different environmental conditions. Maja Škiljo and colleagues observed that during microwave-based soil moisture measurement, a distinct step change appears in the measured data when the moisture content reaches 20% [27]. The resonance frequency of the antenna shifts to a lower frequency for moisture contents ranging from 20% to 100%. the result of a change in the dielectric constant and the loss tangent of the substrate material caused by the presence of moisture [28]. Accordingly, in the present experiment, the moisture content of the material under test was controlled within the range of 0% to 20%.

To improve the accuracy of phase measurement, an experimental calibration of the AD8302-based phase measurement chain was performed. The calibration was carried out at 915 MHz and 5 °C using a precision phase shifter (The phase shifter JSPHS-150+ was sourced from Mini-Circuits, Brooklyn, NY, USA). A total of 19 discrete phase steps from 0° to 180° were applied in 10° increments. The phase shifter output was connected to the phase comparator input of the AD8302, and the corresponding output voltages were recorded. The experimentally measured voltage–phase curve was compared with the ideal curve from the datasheet to establish a calibration function.

A third-order polynomial was fitted to the measured voltage–phase data, yielding the following calibration function:

(15) $$V_{mean}\left(\theta \right)=1.7976-0.01012\theta -1.85\times 10^{-5}\theta^{2}-4.2\times 10^{-8}\theta^{3}$$

where θ is the phase shift in degrees and $V_{mean}$ is the corresponding output voltage in volts. The coefficient of determination $R^{2}=0$.99998 indicates an excellent fit. After calibration, the residual error was reduced to within ±0.015 V, corresponding to a phase measurement uncertainty of approximately ±1.5°.

**Figure 14:**
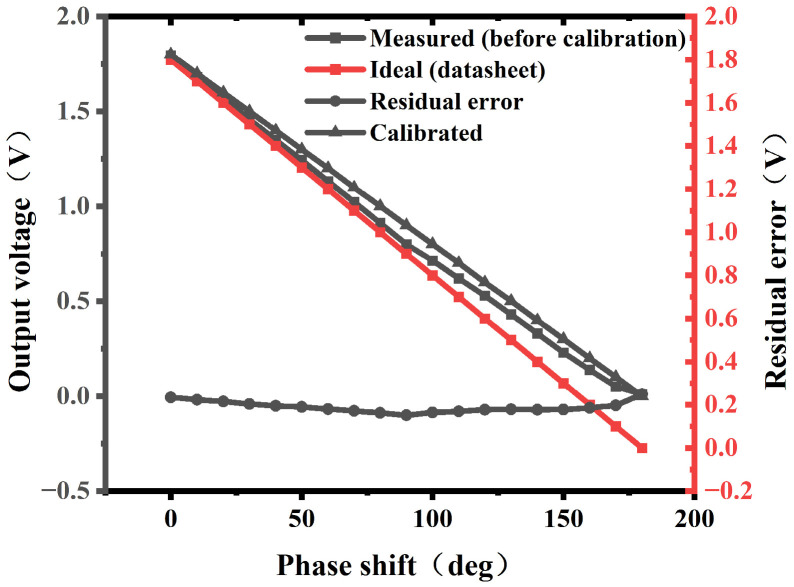
Experimental calibration of the AD8302 phase measurement chain at 915 MHz and 5 °C. The measured data (symbols) are compared with the ideal characteristic from the datasheet (dashed line). A third-order polynomial was fitted to the measured data as the calibration function (solid line). The residual error (right *Y*-axis) is reduced to within ±0.015 V after calibration.

The calibrated voltage–phase relationship is used for all subsequent moisture detection measurements. Figure 14 presents the experimental calibration results of the AD8302-based phase measurement chain at 915 MHz and 25 °C.

**Figure 15:**
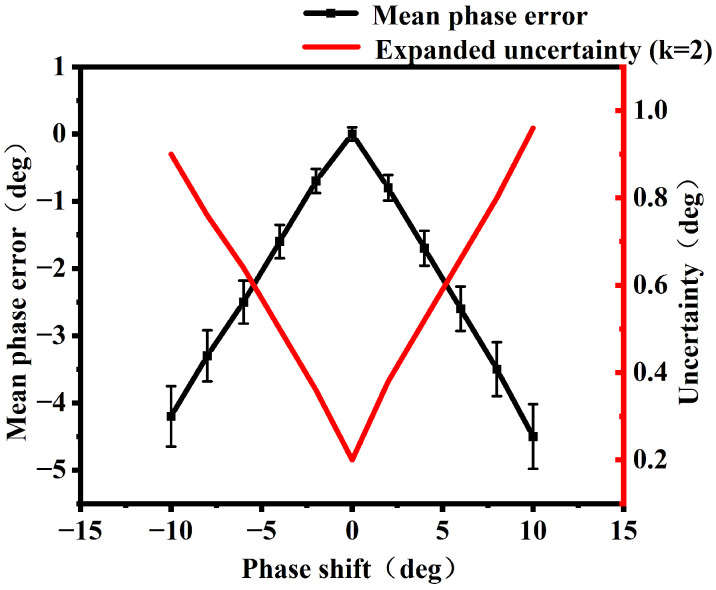
The effect of input power imbalance on phase measurement error at 915 MHz and 25 °C. The mean phase error increases approximately linearly with power imbalance, reaching ±4.5° at ±10 dB, with a slope of approximately 0.4–0.5°/dB. The error bars represent the standard deviation of 50 repeated measurements. The expanded measurement uncertainty (k = 2), shown on the right *Y*-axis, ranges from 0.2° to 0.96°. To maintain the phase error within ±1°, the input power imbalance should be controlled within ± 2.5 dB.

The effect of input power imbalance on phase measurement accuracy was evaluated. At 915 MHz with a fixed 90° phase difference, the power difference between the two input signals of the AD8302 was varied from −10 dB to +10 dB in 2 dB steps. At each imbalance level, 50 repeated measurements were performed, and the resulting phase errors were recorded. As shown in Figure 15, the mean phase error increases approximately linearly with power imbalance, reaching ±4.5° at ±10 dB with a slope of approximately 0.4–0.5°/dB. The expanded measurement uncertainty (k = 2) ranges from 0.2° to 0.96° over the imbalance range. The slight asymmetry of the error curve is likely attributable to the different compression characteristics of the two logarithmic detectors internal to the AD8302. To maintain the phase error within ±1°, the input power imbalance should be controlled within ±2.5 dB.

This requirement is directly relevant to the proposed system. As demonstrated in Section 3.3, the broadband antenna exhibits a radiation efficiency variation of only approximately 2 dB (from −1.0 dB to −3.0 dB) across the 0% to 20% moisture range, which falls well within the allowable imbalance tolerance. Consequently, the phase error caused by power imbalance is effectively suppressed. In contrast, the narrowband antenna shows a severe degradation in radiation efficiency to −12.5 dB at 20% moisture content, far exceeding the ±2.5 dB tolerance and resulting in significant phase measurement errors. These results further confirm that the broadband antenna design not only enhances phase sensitivity but also improves robustness against power-imbalance-induced errors.

**Figure 16:**
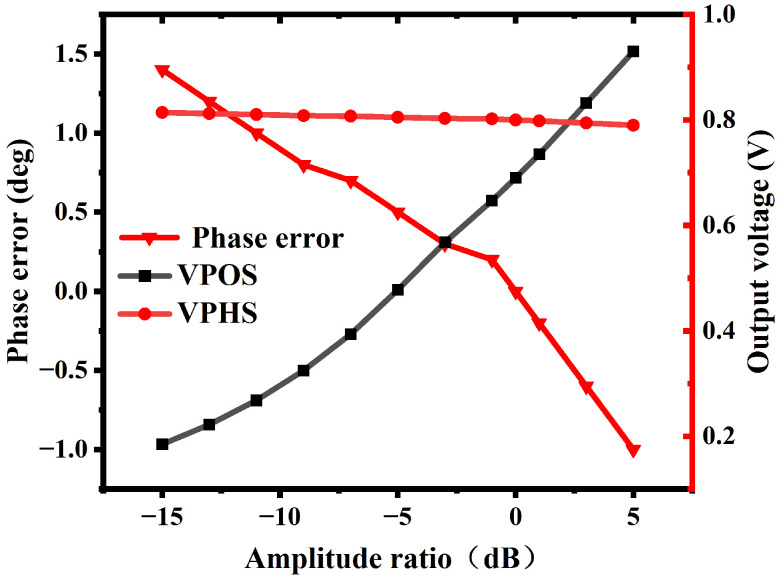
Amplitude-to-phase error evaluation of the AD8302 at 915 MHz and 25 °C. The amplitude ratio between the two input signals was varied from −15 dB to +5 dB with a fixed 90° phase difference. The left *Y*-axis shows the resulting phase error, while the right *Y*-axis shows VPOS and VPHS outputs. The amplitude-to-phase coupling coefficient is approximately 0.08–0.12°/dB.

The cross-interference of amplitude variations on phase measurement was evaluated. Ideally, the amplitude output (VPOS) and phase output (VPHS) of the AD8302 should be independent of each other. In practice, however, due to the non-ideal characteristics of the internal logarithmic detectors, amplitude variations in the input signals can partially couple into the phase output, introducing additional phase measurement errors. The amplitude-to-phase error is defined to quantify this coupling effect. To evaluate this error, the reference channel input power was fixed at 0 dBm, while the measurement channel input power was swept from −15 dBm to +5 dBm in 2 dB steps at 915 MHz with a fixed 90° phase difference, resulting in an amplitude ratio variation from −15 dB to +5 dB. The corresponding VPOS and VPHS outputs were recorded at each amplitude ratio level. As shown in Figure 16, over the amplitude ratio range of −15 dB to +5 dB, VPOS varies from 0.185 V to 0.930 V, while VPHS only changes from 0.814 V to 0.790 V, corresponding to a phase error of −1.0° to +1.4°. These results demonstrate that the amplitude-to-phase coupling is relatively weak, with an amplitude–phase coupling coefficient of approximately 0.08–0.12°/dB. In the proposed moisture detection system, the broadband antenna exhibits a reflected power variation of only about 2 dB across the 0% to 20% moisture range, resulting in a phase error of less than 0.3°, which is negligible.

It should be noted that temperature drift compensation of the AD8302 phase measurement chain has not been included in the current study. This aspect, together with a complete metrological characterization, will be systematically addressed in our future work.

### 4.2. Microwave Moisture Detection Experiment

**Figure 17:**
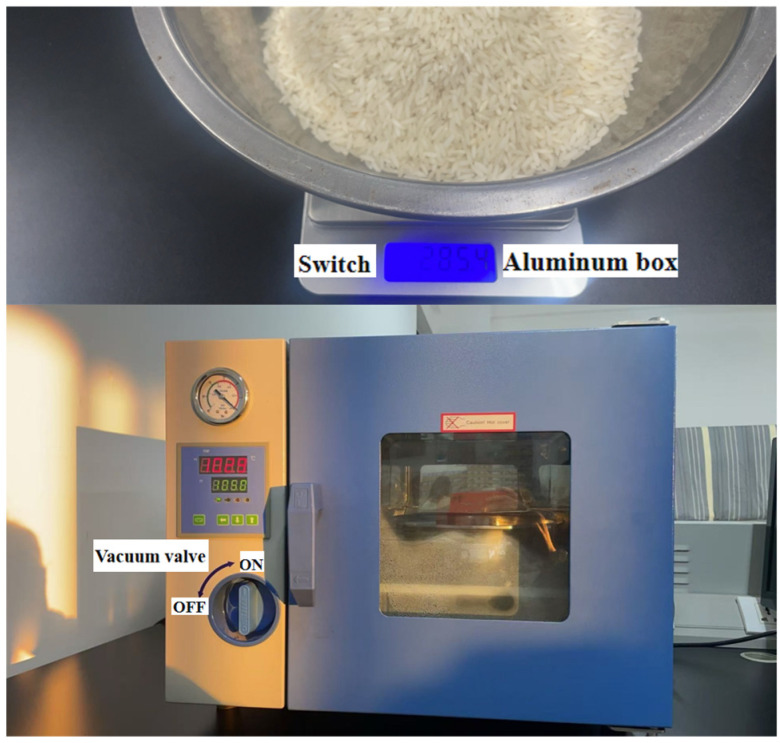
Schematic diagram of material preparation and oven drying.

To make the experimental test data more practical, rice was selected as the test material in this study. The rice originated from Zhangzhou City, Fujian Province. Before the experiment, the rice needed to be husked, and the rice grains were simply screened. Grains that were plump, free from mold, uniform in color, free from disease spots, and intact were selected as the moisture test material. The first step of the experiment was to accurately measure the moisture content of the tested rice. The measurement method followed the relevant provisions of the national standard [29]. According to this standard, when the moisture content of grain exceeds 18%, the two-stage drying method shall be used to measure the moisture content of rice. In accordance with the standard, three samples of rice were taken for each moisture level, with each sample weighing 300 g. The moisture content of each sample was measured separately, and the final measured value was taken as the average of the three measurements. During the drying process, the rice was placed in aluminum boxes with dry inner and outer surfaces. The boxes were then placed in a drying oven and dried at a constant temperature following the relevant provisions of GB/T5497-1985, as shown in Figure 17, until the mass of the rice remained constant.

The true moisture content of the tested rice sample was calculated according to Equation (15) as

(16) $$\rho =\frac{W\times W_{2}-W_{1}\times W_{3}}{W\times W_{2}}\times 100\%$$

where: *W* is the mass of the sample before the first drying, in grams (g);

*W*_1_ is the mass of the sample after the first drying, in grams (g);

*W*_2_ is the mass of the sample before the second drying, in grams (g);

*W*_3_ is the mass of the sample after the second drying, in grams (g).

It is noted that the calculated results of the three rice samples for each moisture level shall not deviate by more than 0.2%. Once this condition is met, the average of the three values is taken as the accurate moisture content of the sample, with the result rounded to one decimal place. Take 300 g of the rice whose moisture content has been measured and evenly place it into a container with dimensions of 150 mm × 150 mm and a height of 100 mm. Cover the sides and bottom of the container with wave-absorbing foam material and position it beneath the antenna. Place the setup in the hardware system for moisture detection to evaluate the system performance.

**Figure 18:**
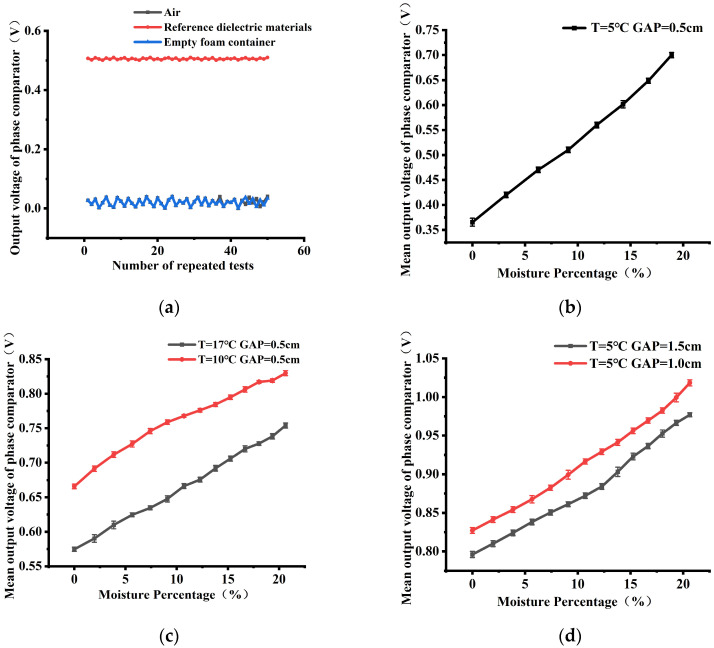
(**a**) The voltage output curves under air, metallic reflector, and empty box environments. (**b**) Moisture content–output voltage curve under initial conditions. (**c**) The moisture content–output voltage curves were repeatedly tested under varying ambient temperatures to evaluate antenna performance. (**d**) The moisture content–output voltage curves were repeatedly tested at varying antenna-to-sample distances to evaluate antenna performance.

During the experimental phase, the experimental platform was first calibrated. As shown in Figure 18a, the moisture measurement system and the antenna were placed in an air environment and tested repeatedly 50 times. The output voltage of the AD8302 fluctuated around 0 V. Combined with Figure 13, it can be concluded that there is no phase difference between the signal received by the antenna and the original signal obtained through the delay cable. When a metal plate was placed beneath the antenna, the output voltage of the AD8302 fluctuated around 0.5 V. Introducing an undesigned metal reflector into the near-field region of the antenna significantly changes the antenna’s input impedance and radiation efficiency through strong capacitive coupling. When the metal object is positioned within 0.1λ below the antenna, the reflection coefficient S11 may fluctuate by more than 10 dB, preventing effective coupling of the transmitted power to the sample under test and making the measurement results extremely sensitive to variations in the distance between the metal object and the antenna. This sensitivity directly undermines the repeatability of the moisture detection system, rendering the calibration relationship between the sample moisture content and the phase difference of the detection signal invalid. Therefore, in practical sensor integration, it is necessary to maintain a sufficient distance (>0.5λ) between the antenna and any underlying metal components, or to use electromagnetic wave-absorbing materials for isolation. In the test environment of this system, while avoiding the presence of metal reflectors within a distance of 0.5λ, foam absorbing material was introduced to wrap the box containing the sample to be measured. The empty box was then placed beneath the antenna and tested repeatedly 50 times. The measurement results returned to fluctuations around 0 V, indicating no phase difference between the signal received by the antenna and the original signal, thus completing the platform calibration.

To better evaluate the detection performance of the novel microstrip antenna, this paper places the antenna under two different environmental conditions for moisture detection. During the experimental phase, the detection performance of the novel microstrip antenna for moisture content was first tested under varying temperature conditions. The ambient temperature was set to 5 °C, and the distance between the antenna and the sample under test was 0.5 cm. Before testing, it was ensured that the no-load voltage output was 0 V. Moisture detection was performed with the moisture content increasing in unit increments, and 40 repeated tests were conducted for each moisture content level. An error bar plot was drawn, as shown in Figure 18b. It can be observed that as the moisture content of the sample increases from 0% to 20%, the mean output voltage of the AD8302 also increases from 0.35 V to 0.7 V, and the standard deviation of the 40 test measurements is extremely small. According to Figure 13, due to the high dielectric constant characteristic of water, a phase change from 145° to 110° is induced. Under the initial environment, changes in moisture content cause significant variations in the voltage output of the phase comparator, and the standard deviation obtained from the 40 repeated tests is extremely small, indicating good stability. A linear fit of the curve yields an R^2^ greater than 0.96, indicating a good linear fit. The test was then repeated under changed ambient temperatures. The ambient temperature was raised to 10 °C and 17 °C, while the distance between the antenna and the sample under test remained at 0.5 cm, and the test method remained unchanged. The resulting two error bar curves are shown in Figure 18c. It can be seen that an increase in temperature does not affect the test performance of the antenna. The standard deviations of the 40 test results are extremely small, and the R^2^ values of the linear fits are all greater than 0.95. Temperature changes affect the slope of the test curve, which satisfies the temperature compensation condition required by Equation (14). This paper verifies the feasibility and stability of the antenna test; the temperature compensation conditions will be supplemented in subsequent research. Since the received power is inversely proportional to the fourth power of the propagation distance, the power decreases sharply as the distance increases. This paper tested the effect of distance on antenna stability. Keeping the ambient temperature constant at 5 °C, two sets of data were tested with the antenna at distances of 0.5 cm and 1 cm from the sample under test. For each moisture gradient, 40 experiments were repeated. The error bar plot is shown in Figure 18d. It can be observed that as the distance changes, the slope of the test curve decreases, which is as expected. However, the R^2^ values of the curve fits calculated from the test results are all greater than 0.95, indicating a good fit. The standard deviation for each moisture gradient calculated from the error bar plot is extremely small, indicating good test stability. Distance compensation is required for the decrease in slope. Since the work in this paper aims to verify the feasibility and stability of the antenna test, the distance compensation method will also be supplemented in subsequent research. After the supplementation is completed, a mature moisture detection system can be launched. It should be noted that while 50 independent repeated measurements demonstrate good short-term repeatability, they do not fully substitute for long-term reproducibility tests under varying environmental conditions. Further validation using different experimental setups is planned as part of our future work.

Considering the antenna performance tests under varying distances and temperatures, it can be observed that the proposed novel microstrip antenna exhibits high sensitivity in capturing the phase shift of the signal caused by the dielectric constant of water in moisture-containing materials. Furthermore, it is capable of performing moisture detection tasks under different environmental conditions, offering advantages such as higher efficiency, better integrability, and lower cost in moisture detection applications. It should be noted that this paper focuses on antenna design and a preliminary feasibility demonstration of the moisture detection concept. A comprehensive metrological characterization, including error bars, repeatability, sensitivity, resolution, limit of detection, and measurement uncertainty, will be systematically addressed in future work.

## 5. Conclusions

In the field of moisture detection, commonly used methods include the drying–weighing method, Karl Fischer titration, near-infrared spectroscopy, and electrical impedance spectroscopy. Microwave-based detection offers advantages such as rapid response, non-contact measurement, and non-destructive testing. However, its application is limited by high cost, and detection accuracy is affected by antenna-induced errors. Microstrip antennas, featuring strong anti-interference capability, simple structure, compact size, and low cost, are well-suited for moisture detection. Nevertheless, their narrow operating bandwidth restricts the effective detection of signal phase changes caused by variations in the dielectric constant of moisture. Although enlarging the antenna size can extend the bandwidth, it compromises the advantage of a compact form factor. Therefore, to develop a novel microstrip antenna that balances both small size and wide bandwidth, this paper first designs a conventional microstrip antenna structure using epoxy resin FR4 as the substrate material. The partial ground plane technique is employed to alleviate its narrowband characteristic, and corner-truncation technology is introduced to extend the operating bandwidth without increasing the antenna footprint. Subsequently, a mathematical relationship model between multiple dimensional parameters and the operating bandwidth is established using the electromagnetic simulation software HFSS. Dimensional optimization is performed with the objective of maximizing the operating bandwidth, ultimately achieving an antenna size of 133 × 125 × 0.5 mm^3^, which maintains a compact form while significantly widening the effective bandwidth. The operating bandwidth is expanded from 0.91–0.92 GHz to 0.6–1.1 GHz, fully meeting the bandwidth requirements for moisture detection. Finally, given that the high dielectric constant of water affects the signal phase, an experimental circuit is designed using the phase comparator AD8302 to detect phase variations, from which the moisture content can be inversely derived. The effectiveness and stability of the antenna for moisture detection are evaluated under different environmental conditions. Experimental results demonstrate that the proposed novel microstrip antenna not only clearly detects signal phase changes induced by moisture content variations but also exhibits high environmental stability, offering a more efficient, stable, and low-cost solution for moisture detection.

## Figures and Tables

**Table 1:** Parameters of the monopole patch antenna.

Parameter	Value
Substrate length $L_{0}$	133 mm
Substrate width $W_{0}$	125 mm
Patch length $L_{P}$	80 mm
Patch width $W_{P}$	72 mm
Microstrip line length $L_{f}$	53 mm
Microstrip line width $W_{f}$	0.63 mm
Partial ground plane $L_{g}$	53 mm
Substrate width h	0.5 mm
Patch length $L_{1}$	50 mm
Patch width $W_{1}$	30 mm

## Data Availability

The original contributions presented in this study are included in the article. Further inquiries can be directed to the corresponding author.

## References

[1] Yu P., Zhu W., Shen C., Qiao Y., Zhang W., Zhu Y., Gong J., Cai J. (2025). Current Status of Grain Drying Technology and Equipment Development: A Review. Foods.

[2] Fields P., Korunic Z. (2000). The effect of grain moisture content and temperature on the efficacy of diatomaceous earths from different geographical locations against stored-product beetles. J. Stored Prod. Res..

[3] Zheng J. (2023). Promoting Agricultural Modernization with New Quality Productivity: Theoretical Logic and Development Path. Price Theory Pract..

[4] Nirmaan A.M.C., Prasantha B.D.R., Peiris B.L. (2020). Comparison of microwave drying and oven-drying techniques for moisture determination of three paddy (*Oryza sativa* L.) varieties. Chem. Biol. Technol. Agric..

[5] Radchenko O.B., Radchenko D.S., Konovets A.I., Grygorenko O.O. (2022). Water Determination in Aromatic Sulfonyl Chlorides Using the Karl Fischer Titration Method: Scope and Limitations. ChemistrySelect.

[6] Ye Y., Shen S., Guo P., Xu X., Wan C., Xu Z. (2022). A Portable Halbach NMR Sensor for Detecting the Moisture Content of Soybeans. IEEE Trans. Instrum. Meas..

[7] Yu L., Zhang M., Yang D., Loescher L., Soleimani M. (2024). Grain Moisture Sensing Using Electrical Capacitance Tomography. IEEE Sens. J..

[8] Wang B., Huang Y. Design and application study of flexible wearable microstrip antenna sensor. Proceedings of the 2021 36th Youth Academic Annual Conference of Chinese Association of Automation (YAC).

[9] Cui Z., Park S., Choo H., Jung K.-Y. (2020). Wideband UHF Antenna for Partial Discharge Detection. Appl. Sci..

[10] Jaruman S., Saman N.M., Xiang H.C., Ahmad M.H., Buntat Z., Adzis Z. Ultra High Frequency Partial Discharge Sensors based on Various Microstrip Patch Antenna Designs. Proceedings of the 2021 3rd International Conference on High Voltage Engineering and Power Systems (ICHVEPS).

[11] Marchal A., Monedero M., Le Thuc P., Staraj R. Ultra-wide band antenna for partial discharge detection inside switchgear for on-line monitoring. Proceedings of the IEEE Conference on Antenna Measurements & Applications.

[12] Hou T. (2023). Research on Wheat Moisture Content Detection Technology Based on Metasurface Plane Wave Antenna. Master’s Thesis.

[13] Liu S. (2021). Research on Microwave Numerical Simulation and Detection Technology for Moisture Content of Stored Wheat. Master‘s Thesis.

[14] Julrat S., Trabelsi S. (2022). Influence of Peanut Orientation on Microwave Sensing of Moisture Content in Cleaned Unshelled Peanuts. IEEE Sens. J..

[15] Lewis M.A., Trabelsi S. (2020). Performance Comparison of Three Density-Independent Calibration Functions for Microwave Moisture Sensing in Unshelled Peanuts during Drying. Appl. Eng. Agric..

[16] Stern F., Taute W., Knöchel R., Höft M. (2023). Dual Antipodal Vivaldi Antenna based Moisture Sensor for Industrial Process Control. IEEE Sens. J..

[17] Julrat S., Trabelsi S. (2019). In-line microwave reflection measurement technique for determining moisture content of biomass material. Biosyst. Eng..

[18] Byron E.V. A new flush mounted antenna for phased-array applications. Proceedings of the 1970 Phased Array Antenna Symposium.

[19] Zhang A., Xu W., Xing D., Zhang Z., Xin D., Gao G. (2022). Design of ultra-wideband monopole partial discharge detection antenna based on meander technology. Electrotech. Appl..

[20] Zhong S. (1991). Microstrip Antenna Theory and Applications.

[21] Abdolrazzaghi M., Daneshmand M., Iyer A.K. (2018). Strongly Enhanced Sensitivity in Planar Microwave Sensors Based on Metamaterial Coupling. IEEE Trans. Microw. Theory Tech..

[22] Kazemi N., Schofield K., Musilek P. (2021). A High-Resolution Reflective Microwave Planar Sensor for Sensing of Vanadium Electrolyte. Sensors.

[23] Abdolrazzaghi M., Katchinskiy N., Elezzabi A.Y., Light P.E., Daneshmand M. (2021). Noninvasive Glucose Sensing in Aqueous Solutions Using an Active Split-Ring Resonator. IEEE Sens. J..

[24] Qin Y., Wei B., Liu S., Li M., Cai C., Hou T. (2021). A New Method for Measuring Complex Relative Permittivity of Dielectric Material by Reflection Method. J. Phys. Conf. Ser..

[25] Varavin A.V., Ermak G.P., Vasiliev A.S., Fateev A.V., Varavin N.V., Zacek F., Zajac J. (2016). Three-Channel Phase Meters Based on the AD8302 and Field Programmable Gate Arrays for Heterodyne Millimeter Wave Interferometer. Telecommun. Radio Eng..

[26] Tu Z.-L., Li E.-K. (2016). Application of AD8302 to Phase Detection System. Shipboard Electronic Countermeasure. https://en.eeworld.com.cn/news/gykz/eic35499.html.

[27] Škiljo M., Blažević Z., Dujić-Rodić L., Perković T., Šolić P. (2022). Self-Sensing Antenna for Soil Moisture: Beacon Approach. Sensors.

[28] Ullah I., Wagih M., Beeby S.P. (2022). Design of Textile Antenna for Moisture Sensing. Eng. Proc..

[29] (1985). Inspection of Grain and Oilseeds—Methods for Determination of Moisture Content.

